# Protective Effects of Quercetin against Dimethoate-Induced Cytotoxicity and Genotoxicity in *Allium sativum* Test

**DOI:** 10.1155/2014/632672

**Published:** 2014-07-21

**Authors:** Waseem Ahmad, Sibhghatulla Shaikh, Nazia Nazam, Mohammad Iqbal Lone

**Affiliations:** Gene-Tox Laboratory, Division of Genetics, Department of Zoology, Aligarh Muslim University, Aligarh, Uttar Pradesh 202002, India

## Abstract

The present investigation was directed to study the possible protective activity of quercetin—a natural antioxidant against dimethoate-induced cyto- and genotoxicity in meristematic cells of *Allium sativum*. So far there is no report on the biological properties of quercetin in plant test systems. Chromosome breaks, multipolar anaphase, stick chromosome, and mitotic activity were undertaken in the current study as markers of cyto- and genotoxicity. Untreated control, quercetin controls (@ 5, 10 and 20 *μ*g/mL for 3 h), and dimethoate exposed groups (@ 100 and 200 *μ*g/mL for 3 h) were maintained. For protection against cytogenotoxicity, the root tip cells treated with dimethoate at 100 and 200 *μ*g/mL for 3 h and quercetin treatment at 5, 10, and 20 *μ*g/mL for 16 h, prior to dimethoate treatment, were undertaken. Quercetin was found to be neither cytotoxic nor genotoxic in *Allium sativum* control at these doses. A significant increase (*P* < 0.05) in chromosomal aberrations was noted in dimethoate treated *Allium*. Pretreatment of *Allium sativum* with quercetin significantly (*P* < 0.05) reduced dimethoate-induced genotoxicity and cytotoxicity in meristematic cells, and these effects were dose dependent. In conclusion, quercetin has a protective role in the abatement of dimethoate-induced cyto- and genotoxicity in the meristematic cells of *Allium sativum* that resides, at least in part, on its antioxidant effects.

## 1. Introduction

Dimethoate (DM) [O,O-dimethyl S-methyl carbamoyl phosphorodithioate] is one of the most important organophosphorus insecticide used extensively on a large number of crops against several pests [1]. For the candidate compound, dimethoate, WHO and US EPA have placed dimethoate in “Toxicity class II,” a moderate toxicant [2, 3]. However, the International Agency for Research on Cancer (IARC) was unable to classify dimethoate with regard to its potential carcinogenicity due to the inadequacies of existing studies [4]. Though these toxicity reports on the hazardous dimethoate are serious enough to warrant a comprehensive documentation of the genotoxic and cytotoxic action on plant test system, the information on the genotoxic properties of dimethoate is limited and inconsistent. Dimethoate is reported to provoke an increase in sister chromatid exchange (SCE) frequency in toadfish lymphocytes* in vitro*, in a concentration-dependent fashion [5] and in mammalian cell cultures [6]. A statistically significant increase in the micronuclei frequency by dimethoate exposure in human lymphocytes was observed in a non-dose-related manner [7] and in bone marrow cells of mammalian system after acute intoxication [8]. In Wistar rats this pesticide was found to increase the incidence of chromosomal aberrations, numerically but not structurally [9]. Contrary to this, dimethoate was found to be negative mutagen in a number of genotoxicity tests [10]. Study of Ündeğer and Başaran proved dimethoate to elicit significant DNA damage in human lymphocytes in the single cell gel electrophoresis [11]. Thus, data on the this compound's genotoxicity are controversial and knowledge on its effect on* Allium sativum* is negligible, to the best of our knowledge, so we aimed in the present study to explore dimethoate genotoxicity, along with the antimutagenic potential of quercetin.

The use of antimutagens and anticarcinogens in everyday life is the most effective procedure for preventing human cancer and genetic ailment. There are several ways in which the action of mutagens can be reduced or avoided. Substances which interfere with DNA repair or with mutagen metabolism can be effective antimutagens [12]. Quercetin—a common flavonoid, is a naturally occurring plant phenolic compound, distributed in many edible fruits and vegetables, and constitutes an integral part of human diet [13] and is considered to be a strong antioxidant due to its ability to scavenge free radicals and bind transition metal ions [14]. It exists in various plants, vegetables and fruits, specifically in red onions, grapes, berries, cherries, broccoli, citrus fruits, tea (*Camellia sinensis*) and capers [15]. Quercetin is able to preclude oxidative stress by directly inactivating free radicals, by xanthine oxidase inhibition and lipid peroxidation, and by affecting antioxidant pathways both* in vivo* and* in vitro* [16, 17].

Quercetin, being a strong anti-oxidant, is renowned scavenger for highly reactive species like hydroxyl radicals and peroxynitrite [18] and superoxide radicals [19]. Consequently, it has been shown to protect against oxidative DNA damage (single strand breaks) in human lymphocytes* in vitro* [20] and sperm [21]. Also in cell lines Caco-2, Hep G2, and V79, quercetin is able to protect against DNA single strand breaks in a direct manner, instead of an increase of repair rate which is known from flavonoids [22]. In a human melanoma cell line (HMB-2) quercetin reduced the frequency of chromosomal aberrations induced by H2O2 [23].

Although the antimutagenic potential of quercetin has been extensively studied and well reported, yet no report exists on the biological effects of quercetin in plant model. The current study is designed to explore the action of quercetin against dimethoate induced chromosomal aberrations in* Allium sativum* root meristem cells.

## 2. Materials and Methods

### 2.1. Test System

The onion (*Allium sativum*, 2*n* = 16) bulbs equal in size 1.5–2.0 cm diameter were chosen from a population of locally available commercial variety, Nasik Red (*N* − 53).

### 2.2. Chemicals

Dimethoate, CAS number 60-51-5, was a product of Sigma. Ethanol (Merck) was of analytical grade. Glacial acetic acid CAS number 64-19-7 and hydrochloric acid were products of Fisher Scientific. Acetocarmine CAS number 64-19-7 was a product of Loba Chemie.

### 2.3. Root Harvest and Slide Preparation

Root tips of 1–3 cm long were cut and divided into four groups. Untreated control, quercetin controls (5, 10, and 20 *μ*g/mL for 3 h), dimethoate treated groups (100 and 200 *μ*g/mL for 3 h), and dimethoate along with quercetin as the last group was treated with different concentrations, 5, 10, and 20 *μ*g/mL of quercetin for 16 h. Following quercetin treatment, the bulbs were washed in distilled water and then treated with 100 and 200 *μ*g/mL of dimethoate for 3 h and placed in a watch glass. The untreated and exposed root tips were fixed in acetic alcohol (ethanol : glacial acetic acid in 3 : 1 ratio) for 12 h at room temperature. After this the root tips were hydrolyzed in 1 N HCL at 60°C for 10 minutes and stained with acetocarmine for 20 minutes and then squashed on glass slide under 45% acetic acid to determine the mitotic index and the presence of chromosomal aberrations.

### 2.4. Microscopic Examination

Three bulbs were used for each dosage. A total of 300 well spread metaphases per bulb were analyzed for chromosomal aberrations and 3000 cells were scored for mitotic index. The mitotic index for cytotoxicity evaluation was calculated by dividing cells out of total cells counted. The suppression percentage (SP) of quercetin on chromosomal aberrations of dimethoate is calculated as [24]

(1) $$\begin{array}{c} \text{SP}\left(\%\right)=100\%-\frac{N1}{N2}\times 100\%, \end{array}$$

where *N*1 is the number of aberrations in quercetin pretreated and dimethoate posttreated groups and *N*2 is the number of aberrations in dimethoate alone treated group.

### 2.5. Statistical Analysis

Data on total number of aberrations, mitotic index, and abnormal metaphases were analyzed by analysis of variance (ANOVA), with the calculations of the* F*-statistic and respective *P* values. The *P* values were compared with calculation of the minimum significant difference for *P* ≤ 0.05%.

## 3. Results

**Figure 1:**
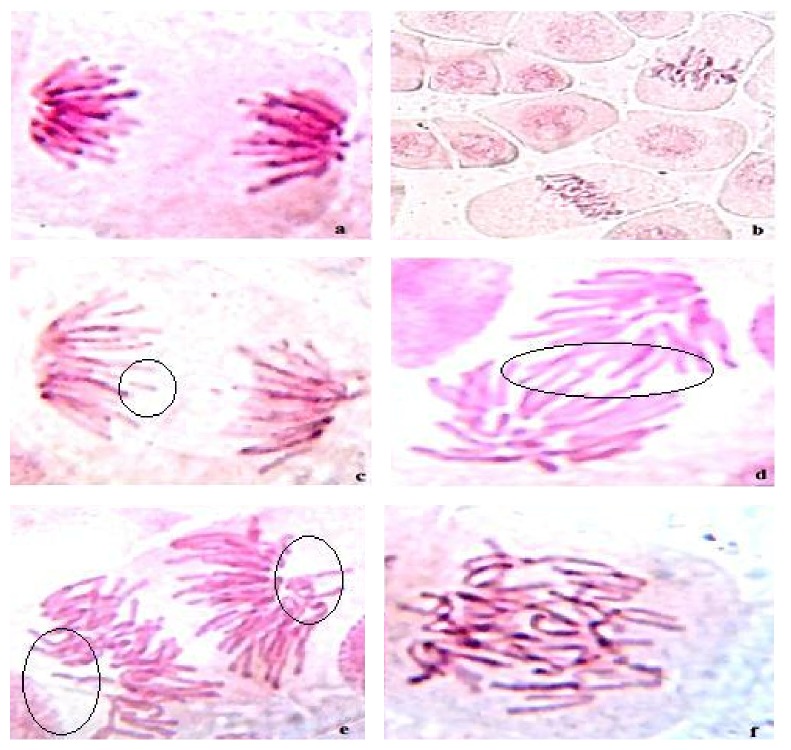
* Allium sativum* root tip cells showing normal anaphase (a) and normal metaphase (b) in negative control group, mitotic abnormalities induced by pesticide treated group showing chromosome break (c), lag chromosome (d), multipolar anaphase (e), and stick chromosome (f).

The representatives of dimethoate induced chromosomal aberrations such as break, lag chromosome, stick chromosome, and multipolar anaphase analyzed in* Allium sativum *root tip cells are shown (Figure 1). Quercetin induced chromosomal aberrations at all the multiple doses, that is, 5, 10, and 20 *μ*g/mL, were not statistically significant when compared with untreated control, which indicated its nonclastogenicity. The number of aberrations and the number of abnormal metaphases induced by dimethoate increased in a dose dependent manner, which represented its mutagenic action in* Allium sativum* and was statistically significant (*P* < 0.05) when compared with untreated control (Table 1). As the concentrations increased, the number of abnormal metaphases and the number of aberrations decreased significantly in all quercetin pretreated groups. The percentage of mitotic indexes decreased with increasing concentrations of quercetin compared with the untreated control, which suggests its cytotoxicity in plant test system. Similarly the reduction of mitotic index was also found in all quercetin pretreated groups, except 5 *μ*g/mL. The percentage of suppression by quercetin on dimethoate induced chromosomal aberrations increased with increasing concentrations of quercetin in all the concentrations tested, indicative of its antimutagenic potential in* Allium sativum*. The effect of quercetin on the reduction of total number of aberrations induced by dimethoate was statistically significant when compared with dimethoate control. This study implies that pretreatment of quercetin has a strong inhibitory role against the mutagenic action of dimethoate.

## 4. Discussion

Plant system is excellent indicator system and provides reliable bioassays for mutagenic studies in higher eukaryote, having a variety of well-defined genetic endpoints including ploidy alteration, chromosomal abnormalities, and SCEs [25]. The clastogenicity of three well-known and widely used herbicides (i.e, pentachlorophenol, 2, 4-D and butachlor) have been reported in our earlier studies using plant test system,* Allium *root tip test [26]. And very recently, the clastogenic and genotoxic potentials of an organochlorine, dichlorophen and an organophosphate, and dichlorvos have been studied using root meristematic cells of* Allium cepa* [27]. To the best of our knowledge, no study has been carried out on the cytotoxic and genotoxic effects of dimethoate on root meristem cells of* Allium sativum*, despite the fact that it is widely used. The result of the present study indicates that dimethoate can induce cytotoxic and genotoxic effects on the meristematic cells of* Allium*. The MI inhibition and induction of chromosomal aberration in plant cells by several pesticides have been reported earlier by different workers [28, 29]. Mitotic activity reduction could be due to inhibition of DNA synthesis [30] or due to a block in the G2-phase of the cell cycle; hence cell is prevented from undergoing mitosis [31]. The mitotic activity suppression is often used to assess cytotoxicity [32].

Several chromosomal aberrations (CA's) like chromosomal break, stickiness, laggard, and multipolar anaphase have been formed. The induction of chromosome breaks by pesticides indicates the clastogenic potential of the test compounds [33]. Chemicals that induce chromosome breakage are known as clastogens and their action on chromosome is generally regarded to involve an action on DNA [34]. Laggards were observed which are due to the failure of the chromosome to move to either of the poles. According to Kaur and Grover, the lagging chromosomes can be attributed to the delayed terminalization and stickiness of chromosomal terminals or due to the collapse of chromosomal movement [35]. Chromosome stickiness was another frequent chromosomal abnormality induced by dimethoate in meristematic cells of* A. sativum*. This stickiness is presumably due to intermingling of chromatin fibers which lead to subchromatid connection between chromosomes [36]. Stickiness can also be explained as physical adhesion of the proteins of the chromosome [37].

In the present study quercetin exhibits antimutagenic potential against dimethoate induced damage in a dose dependent manner. Several authors also reported that quercetin is known as a potent scavenger of free radical species, competent of inhibiting lipid peroxidation in* in vitro* and* in vivo* systems [38, 39]. It has been reported that quercetin is a member of the flavonoid family which can delay oxidant injury and cell death by scavenging ROS and free radicals [40].

The antioxidant activity of quercetin can be explained by its chelating property, since transition metal ions such as the iron ion play a crucial role in the generation of reactive oxygen species. Also, the catechol group is recognized to contribute directly to the chelating action of quercetin [41]. In fact, a number of studies have demonstrated that quercetin inhibits lipid peroxidation effectively by scavenging free radicals and/or chelating transition metal ions [42].

The above mechanism for antimutagenic actions of quercetin was discovered in different test systems apart from plants. For the present study, the mechanism of action is yet unknown. The possible justification for the antimutagenic potential of quercetin in this study may be due to the following: trapping of free radicals, toxic gas degeneration, ion degradation, and peroxide accumulations. However, the exact mechanism by which quercetin protected against dimethoate-induced cyto- and genotoxicity in root meristematic cells is not fully understood. One probable rationalization for the protection against cyto- and genotoxicity is that simultaneous treatment with quercetin would allow interception of free radicals generated by dimethoate before they reach DNA and induce cyto- and genotoxicity.

## 5. Conclusions

On the basis of our results, we conclude that quercetin has antimutagenic potential against dimethoate induced clastogenic damage in* Allium sativum* in a dose dependent manner, but it is more effective at low dose (5 *μ*g/mL). However the mechanism by which it acts remains to be investigated in plant test system and further studies are necessary to clarify this point.

## Figures and Tables

**Table 1:** Distribution of different types of chromosomal aberrations in 300 cells analyzed and mitotic index observed in *Allium sativum* after treatment with quercetin and/not dimethoate.

Treatment	MI	AM	Aberrations					
			Br	LC	SC	MP	Total	SP (%)
Untreated control	8.06	4	2	—	1	—	3	—
QUR (*μ*g/mL)								
5	6.07	3	2	1	—	1	4	—
10	5.23	4	3	1	2	1	7	—
20	4.64	6	5	1	—	1	8	—
DM_1_ 100 (*μ*g/mL)	4.68	32∗∗	23∗∗	5	4	3	35∗∗	—
QUR 5 (*μ*g/mL) + DM_1_	5.02∗	24∗	19∗	5	2	1	27^#^	22.8
QUR 10 (*μ*g/mL) + DM_1_	4.76∗	22∗	18∗	1	2	2	23^#^	34.2
QUR 20 (*μ*g/mL) + DM_1_	3.78∗	19∗	13∗	4	2	2	20^#^	42.8
DM_2_ 200 (*μ*g/mL)	3.94	40∗∗	32∗∗	6	5	3	46∗∗	—
QUR 5 (*μ*g/mL) + DM_2_	4.76∗	28∗	22∗	8	3	2	33^#^	28.3
QUR 10 (*μ*g/mL) + DM_2_	3.46∗	24∗	19∗	4	3	1	28^#^	39.1
QUR 20 (*μ*g/mL) + DM_2_	3.30∗	20∗	17∗	3	2	1	23^#^	50.0

QUR: quercetin; DM: dimethoate; MI: mitotic index; AM: abnormal metaphases; Br: break; LC: lag chromosome; SC: stick chromosome; MP: multipolar anaphase; SP: suppression percentage. ^#^Statistically different when compared with dimethoate control. ∗Statistically different when compared with quercetin control. ∗∗Statically different when compared with untreated control.
